# Polycythemia Vera Presenting As Acute Non-hemorrhagic Infarction and Chronic Bleed: Deception Between the Lines

**DOI:** 10.7759/cureus.61350

**Published:** 2024-05-30

**Authors:** Palash S Kotak, Suprit Malali, M Jayanth Kumar, Sourya Acharya, Sunil Kumar

**Affiliations:** 1 Department of Medicine, Jawaharlal Nehru Medical College, Wardha, IND

**Keywords:** stroke, hematocrit, platelets, hyper-viscous, genetic, jak2

## Abstract

Patients with polycythemia vera (PV) develop various complications due to hyper-viscous blood, causing events such as ischemic stroke. There are other associated complications due to the dysfunction of platelet activity, causing hemorrhages. In our unusual case, we present a patient who came to the OPD complaining of slurring speech. An MRI was done and was suggestive of acute lacunar infarcts with changes in chronic bleed. CBC and hematocrit were consistent for PV, with the genetic marker JAK2 being positive.

## Introduction

The myeloproliferative disease polycythemia vera (PV) is usually linked to a gain of function mutation in JAK2 617V [1]. This disorder primarily involves hematopoietic stem cells, leading to excessive erythrocytosis [2]. The increased erythrocyte production causes increased viscosity, leading to a multitude of complications such as thrombosis, stroke, myocardial ischemia, deep vein thrombosis, pulmonary embolism, etc. [3-5]. PV is uncommonly associated with coagulopathy due to dysfunction in platelet aggregation, causing increased activated partial prothrombin time [3]. In around 15% of cases of PV, it initially presents as a stroke [6]. Here, we present a case of acute lacunar infarct with chronic bleed in a 52-year-old male. After the patient came to us with symptoms of stroke, initially, we attributed the stroke to hypertension. However, later on, we were able to diagnose PV as the cause of the same.

## Case presentation

**Figure 1:**
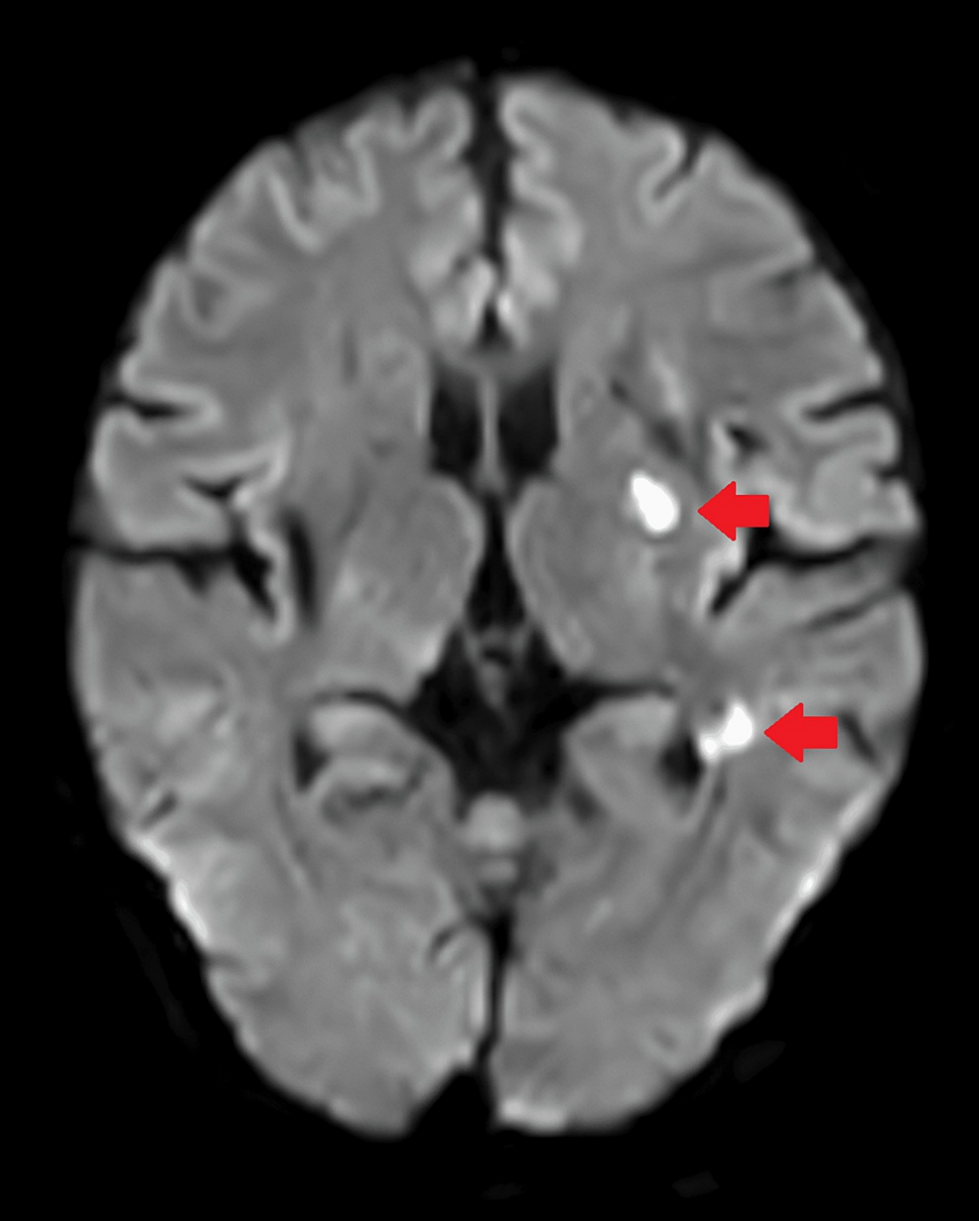
Diffusion-weighted image showing two non-hemorrhagic infarcts in the left corona radiata

**Figure 2:**
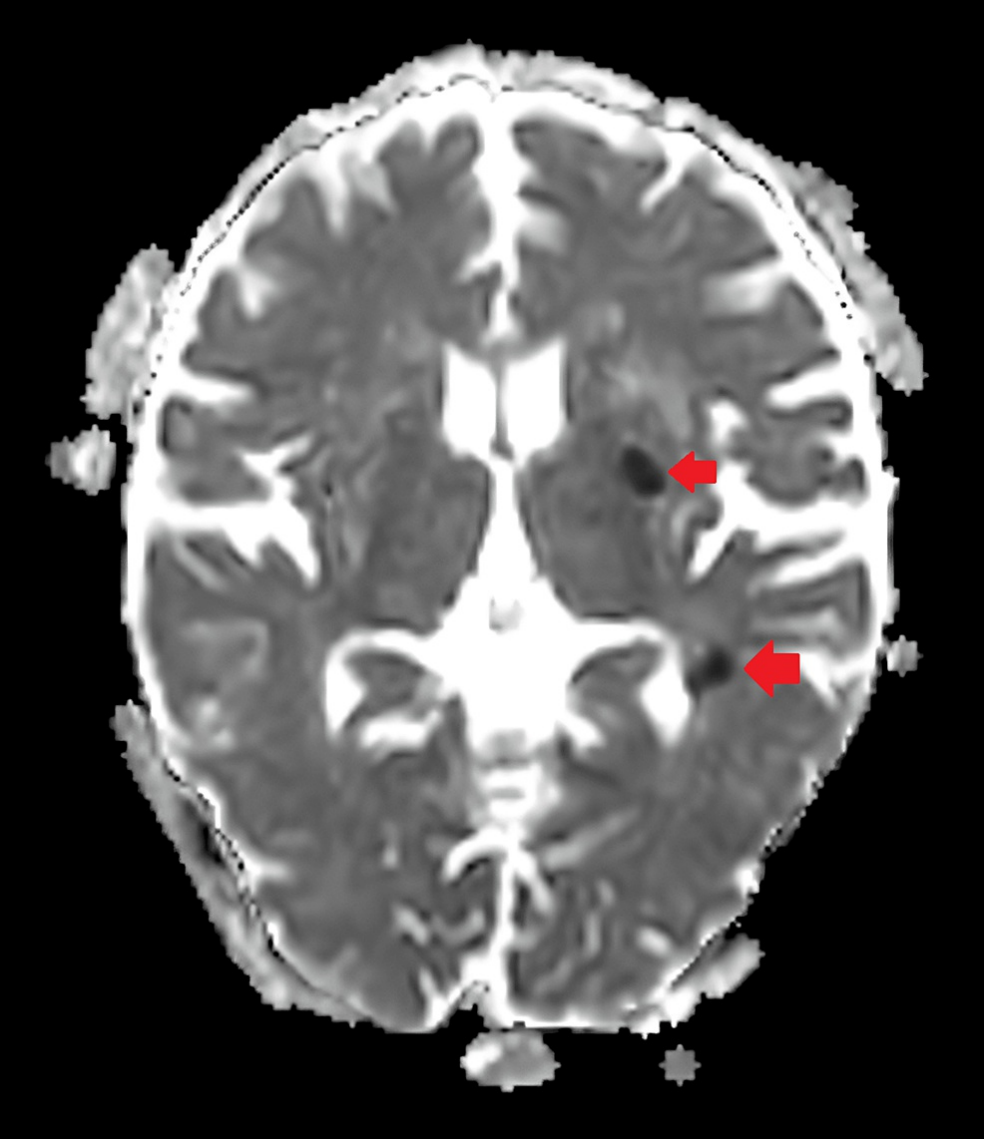
Findings of diffusion-weighted imaging confirmed which appears as low signals in the apparent diffusion coefficient

**Figure 3:**
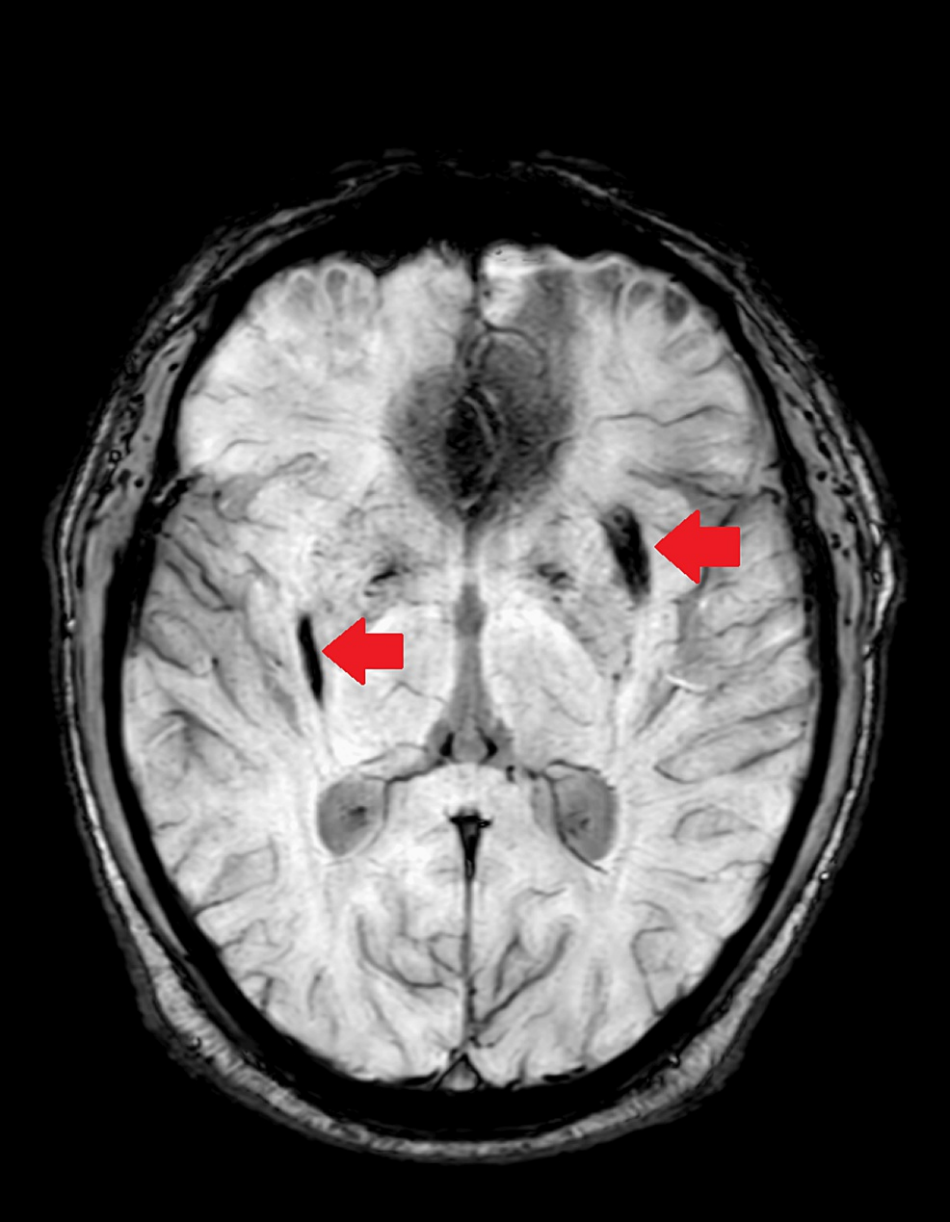
Susceptibility-weighted image showing chronic bleed in the bilateral gangliocapsular region

A 52-year-old male patient arrived at the OPD with chief complaints of slurring speech, which was associated with deviation of the angle of the mouth for two days. He had a previous history of hypertension and was taking a combination of amlodipine (5 mg) and atenolol (50 mg) once a day. On physical examination, his pulse was 74/minute, and his BP was 120/70 mm Hg. On central nervous system examination, there was right-sided supranuclear paralysis with a deviation of the tongue to the right side, and plantars were extensor. An MRI of the brain was done, which revealed two acute non-hemorrhagic lacunar infarcts in the left corona radiata, gangliocapsular region, and left temporal lobe. A chronic bleed was noted in the bilateral gangliocapsular region (Figures 1-3).

**Figure 4:**
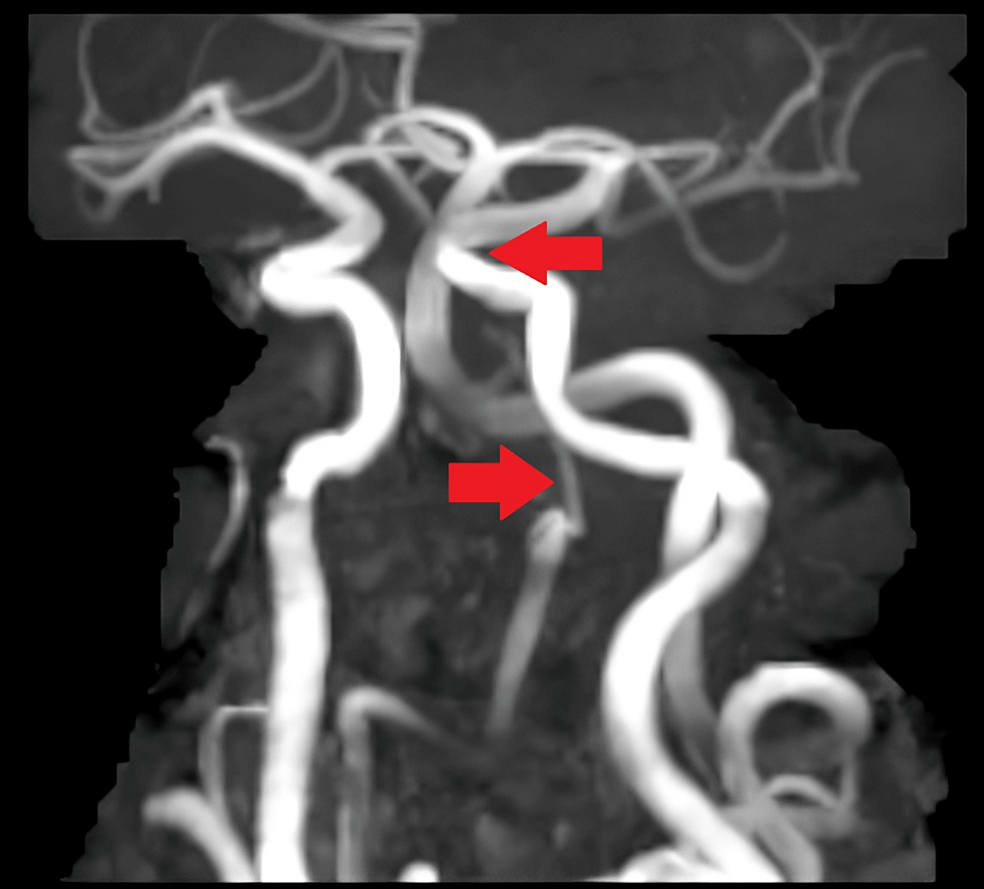
Kinking is noted at the vertebro-basilar junction and the basilar artery without luminal compromise. The bilateral common carotid artery, internal carotid artery, external carotid artery, circle of Willis, and its branches appear normal in course and caliber

**Figure 5:**
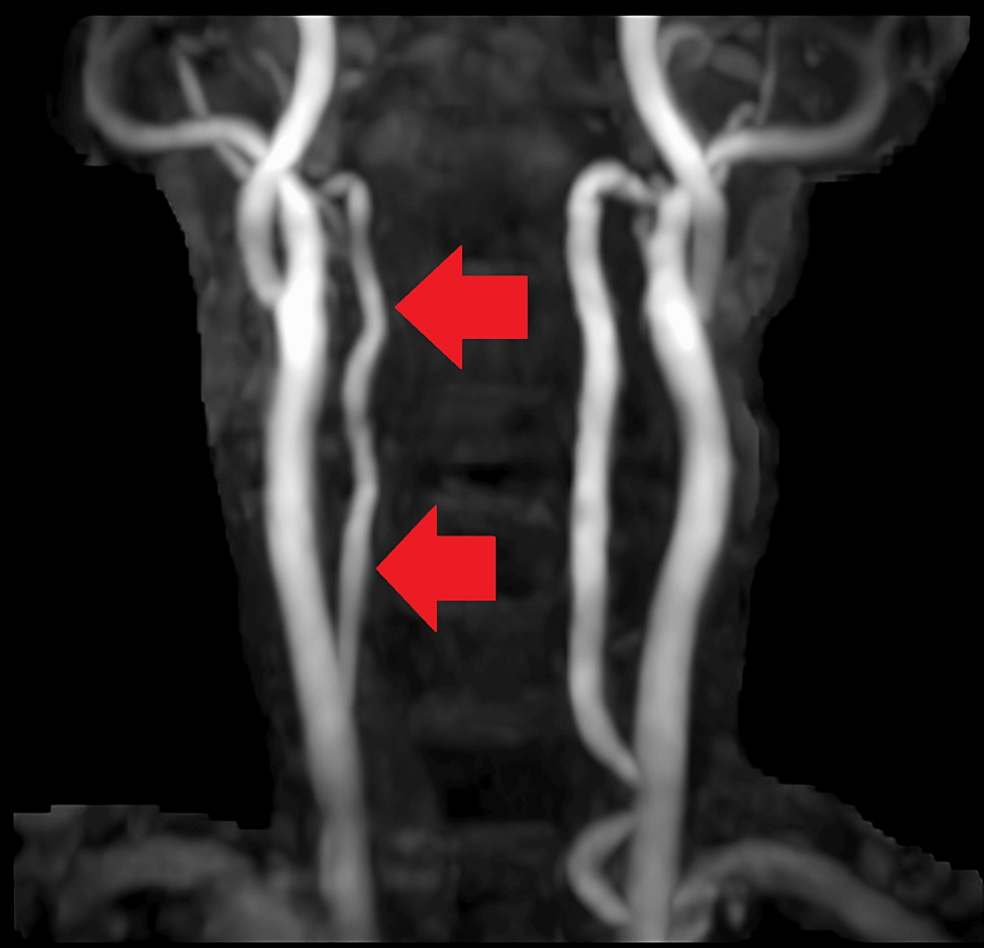
The right vertebral artery is hypoplastic

The MR angiography suggested a hypoplastic right vertebral artery with the left vertebral artery continuing as the basilar artery and kinking at the vertebra-basilar junction and basilar artery without luminal impairment (Figures 4-5).

A routine workup revealed his hemoglobin to be 18.3 gm/dL, his leucocyte count to be 13100/mm^3^, and his platelets to be 2.77 x 10^5^/mm3. His hematocrit was 53.3%. The possibility of PV was considered. To confirm the same, the patient was taken up for a genetic study for PV. The reports were positive for JAK2 617V, and his erythropoietin was low (2.49 mIU/mL). All the relevant investigations are summarized in Table 1, given below.

**Table 1:** Observed blood investigations in our patient

Serial Number	Investigation	Reference Range	Observed Value
1	Hemoglobin	13-17 gm/dL	18.3 gm/dL
2	Hematocrit	40-50%	53.3%
3	Platelet	1.50-4 .10 x 10^5^/mm^3^	2.77 x 10^5^/mm^3^
4	Leucocyte counts	4000-10000/mm^3^	13100/mm^3^
5	Erythropoietin	5-35 mIU/mL	2.49 mIU/mL
6	JAK2 617V	Negative	Positive

To improve his symptoms, the patient was started on aspirin and a therapeutic phlebotomy of around 330 ml. Further, he was advised to do CBC every 15 days, and therapeutic phlebotomy would be done only when hematocrit levels were >45% to achieve target hematocrit levels of <45%. Additionally, he was started on a tab of hydroxyurea 500 mg once a day along with low-dose aspirin 75 mg once every day. Once the patient's symptoms improved, he was discharged and advised to follow up.

## Discussion

The myeloproliferative neoplasm linked to constitutive activation in the signal transduction pathway of JAK-STAT is known as PV [1]. Mutation in JAK2 617V or other JAK2 exon 12 regions is typically observed in 95% of the cases [1].

Several mechanisms, including endothelial injury and its subsequent interaction with activated platelets, are implicated in thrombosis secondary to PV [3,4]. Apart from factors relating to platelets, a high erythrocyte volume can cause blood to become more viscous, and blood flow might be hindered, which can result in vascular consequences such as peripheral ischemia, transient ischemic attacks, ischemic infarcts, and erythromelalgia [3-5]. Our case was a regular follow-up patient for hypertension, and he was compliant with medications. His BP records show tight control of his BP. Also, no atherosclerotic plugs or carotid stenosis were seen in the patient's MR angiography. Hence, ischemic infarcts infarct and chronic bleed were attributed to PV and not hypertension.

The World Health Organization updated its diagnostic standards for PV in 2016 [7]. There are now three major and one minor criteria in the diagnostic criteria for PV [7]. The WHO PV standard is as follows.

The major criteria are hematocrit levels >49% in males and >48% in females, hemoglobin levels >16.5 g/dL in males and >16.0 g/dL in females, and an increase in red cell mass >25% over the mean standard predicted value. The second significant criteria include the results of the bone marrow biopsy, which show substantial proliferation of erythroid, granulocytic, and megakaryocytic cells as well as pleomorphic adult megakaryocytes (differences in size), trilineage growth (panmyelosis), and hypercellularity for the patient's age. Furthermore, the third main criterion is the existence of either the JAK2 exon 12 mutation or JAK2V617F. A low or subnormal serum erythropoietin level is a minor criterion.

PV diagnosis needs fulfillment of the first main criterion in the presence of the third main criterion, the minor criterion, or all three major requirements [7]. Treatment modalities primarily aim to bring the hematocrit values to an acceptable range with cytoreduction and reduce the prevalence of thrombotic events. A target of <45% is associated with improved morbidity and mortality in PV subjects [4]. Phlebotomy is used in conjunction with hydroxyurea chemotherapy and anagrelide medicine, preventing platelets from maturing [4,8]. Antiplatelet therapy with low-dose aspirin is known to cause an overall reduction in mortality and thrombotic events [9,10]. In our instance, therapeutic phlebotomy was used to reach goal hematocrit levels of less than 45% when hematocrit levels were >45%. He was also started on low-dose aspirin 75 mg once daily in addition to hydroxyurea 500 mg once daily to improve his prognosis. In our case, PV unexpectedly caused the ischemic stroke, which can have various underlying factors [6,11].

## Conclusions

Patients with PV have a multitude of factors contributing to various strokes. The treatment modality of choice remains phlebotomy to achieve the target hematocrit levels. Adjuvant chemotherapy using hydroxyurea is also needed. Antiplatelet therapy with aspirin is also added in these patients to improve the overall morbidity and mortality secondary to thrombotic events.
